# Patients offered genomic testing for rare disease and cancer: a real-world evaluation of impact and processes of care

**DOI:** 10.1038/s41431-026-02138-2

**Published:** 2026-05-19

**Authors:** Melissa Martyn, Ling Lee, Emily Forbes, Anaita Kanga-Parabia, Callum McEwan, Rigan Tytherleigh, Alli Jan, Ella Lynch, Ivan Macciocca, Clara Gaff

**Affiliations:** 1 Melbourne Genomics Health Alliance, Walter and Eliza Hall Institute of Medical Research, Melbourne, VIC Australia; 2 Department of Paediatrics, The University of Melbourne, Melbourne, VIC Australia; 3 Murdoch Children’s Research Institute, Parkville, MO Australia; 4 Victorian Clinical Genetics Services, Parkville, MO Australia

**Keywords:** Health policy, Translational research, Outcomes research, Genomics

## Abstract

Robust evidence is required to support decision-making about incorporating genomics into healthcare; patient perspectives are crucial. Prior studies centre on people giving research consent for testing, yet significant differences between research and clinical cohorts are well established. We investigated 1690 patients offered genomic testing during clinical care by a range of medical specialists, for rare diseases and cancer. Ninety per cent (1515) accepted testing. Of 74 decliners providing their reasons, 20 gave genomic-specific concerns. Impact and experiences of care were captured using surveys after consent (S1:RR 73%) and return of results (S2:RR 53%). We actively included those often missing from research, e.g. 8% of S2 respondents accepted telephone assistance - typically with interpreters - to complete surveys. Those who spoke English as an additional language were less likely to have received enough information at pre-test counselling (88% v 96%) and less likely to correctly answer questions about potential genomic test results. After receiving results, 10% (52/534) of respondents had moderate-high decision regret; predictors included English as an additional language and not receiving enough information at consent. Value from testing was quantified and compared: those with informative results valued their medical and personal utility; those with uninformative results derived social utility. Perceived personal control increased post-result for those with diagnostic results and decreased for those with uninformative results. Our results expand the evidence base available for genomic health technology assessment. On balance, genomic test results provide more value than harm, but equity issues need to be addressed to ensure all patients can benefit.

## Introduction

A key challenge in integrating genomics into healthcare systems is evidence—for when genomics testing does (and does not) offer advantages and how to provide it in routine care, particularly beyond clinical genetics [1, 2]. To ensure safe, high-quality care, this evidence must include patient perspectives on value and processes of care [3–5]. To maximise utility for decision-makers at national, state and hospital policy levels, such evidence should be obtained as close to real-world care as possible [6] and should capture diverse patient perspectives. Evaluative research to date in genomic medicine focuses on cohorts offered research genomic testing. However, research participants are often less diverse than the population [7] and research protocols differ from actual healthcare processes. The more they deviate from these, the less useful the results are for informing healthcare processes.

Achieving quality, high-value care for patients should be the overarching goal of healthcare delivery. Acceptability of genomic testing offered under research governance has been explored [7] but information on why patients decline clinically indicated genomic testing is lacking. Previous studies of participant experience of pre- and post-test genetic counselling have found that a majority report making informed decisions [7], had low decision regret [8] and found pre-test counselling helpful [9]. However, these findings are from large genomic sequencing initiatives using research consent for genomic testing and capturing participant perspectives in surveys only in the primary language of the study. The experiences of diverse patient groups with clinical care processes around genomic testing are not known.

It is widely recognised that genomic testing can provide value to patients that extends beyond narrowly defined ‘medical utility’ [10–12]. Non-health value includes personal dimensions such as emotional, planning and behavioural value, as well as ‘social’ value, from contributing to research [10–12]. Limited qualitative evidence suggests an influence of test outcomes on realised value [11–13] but this has not been clearly established. The academic literature [10, 14, 15] and health technology assessment guidelines [16] recognise the importance of including non-health value in genomic testing assessment. A standardised approach to capture personal utility of research testing has been developed [17], but the challenge of how to quantitatively capture the wide range of value from clinical testing to inform funding decisions remains [15].

To inform the design of care processes that support high-quality care and do not exacerbate existing inequities, it is necessary to understand diverse patient experiences of genomic testing. Evaluation of the patient experience within the Melbourne Genomics Health Alliance program was designed to inform decision-making by governments and health services. After an initial demonstration phase [6], genomic testing was provided prospectively in clinical care, under clinical governance in most cases. Patient perspectives were captured in both impact and process evaluations, which were informed by consumer voices [18]. We made it a priority to include the voices of patients often missing from research studies—those unable through language, literacy or disability to complete a written survey—by providing telephone assistance, with interpreter support as needed, for survey completion. The Melbourne Genomics program funded clinical testing for patients with a wide range of conditions spanning cancer and rare disease; we attempted to harmonize approaches to measure value across these diverse conditions, using existing PROMs and novel approaches. The clinical impacts of genomic testing of patients within this initiative have been reported elsewhere (see references in Table 1). Here, we explore the impact of genomic testing reported by patients, including the value realised by those with informative and uninformative results (impact evaluation) and patient experiences of the processes of care. As one of the few programs [19] including patients having somatic genomic testing for cancer and those with rare disease, we are able to compare experiences of these two groups and investigate how well the measures we used performed across these different indications. Importantly, we compare the experiences of those who are rarely, if ever, included in genomic studies with those who are well represented.

**Table 1:** Acceptance of testing across clinical service design projects.

Project	Clinical setting	Testing uptake [accepted/offered (%)]^a^
***Rare disease projects***		
Complex Care [47–49]	Clinical Genetics (paediatric)	137/144 (95)
Complex Neurological and Neurodegenerative Diseases [50]	Adult neurology	161/204 (79)
Deafness [51]	General paediatrics	106/169 (63)
Dilated Cardiomyopathy [52]	Adult and paediatric cardiology	94/100 (94)
Immunology [53]	Adult and paediatric immunology	198/208 (95)
Renal Genetics [31]	Renal Genetics	204/225 (91)
Perinatal autopsy	Perinatal pathology & Clinical genetics	67/80 (84)
***Haematological/Malignant projects***		
Bone Marrow Failure (BMF) [54]	Adult and paediatric haematology	116/118 (98)
Non-Hodgkin Lymphoma^b^	Adult haematology	117/123 (95)
Solid Cancers [20]	Adult and paediatric oncology	315/319 (99)
Total		1515/1690 (90)

^a^No response received from 18 patients and 15 patients offered testing were deemed ineligible.

^b^Research consent for testing provided by participants in this project.

## Patients and methods

### Study design and setting

This is an evaluation study of patient experience of being offered genomic testing funded through the Melbourne Genomics Health Alliance program. This study has Human Research Ethics Committee approval from the Royal Melbourne Hospital Human Research Ethics Committee (HRC/13/MH/326). For nine of the 10 clinical projects, consent for genomic testing was clinical, rather than research consent. All participants provided informed consent for participation in research activities. Consent for genomic testing and consent for participation in evaluation research were obtained between March 2016 and September 2018.

### Participants, pre-and post-test counselling and consent

Between 2016 and 2020, Melbourne Genomics Health Alliance evaluated diagnostic and treatment-directed genomic testing for adults and children for a range of indications spanning rare disease and cancer [6]. Patients were offered genomic testing as part of their clinical care through one of 10 clinical service design projects. Projects operated across the five member hospitals, with some also accepting referrals from clinicians in the private sector. In nine projects, surveys were used to capture patient experiences of testing (Supplementary Table 1).

Those with rare diseases were offered exome sequencing with targeted analysis. Patients in the solid cancer project were offered a large ( ~ 400 gene) panel. Patients in the haematological projects (bone marrow failure and non-Hodgkin lymphoma) were offered targeted panel and exome testing where clinically relevant. Testing was raised by treating clinicians. Pre- and post-test genetic counselling was provided by genetic counsellors for all indications except solid cancers [20], where a medical oncologist with genomics expertise provided pre-test counselling and disclosed results. As per usual clinical practice, genetic counselling was provided in English, with interpreters used when required.

### Data collection—clinical care

Patients’ decisions to accept or decline genomic testing were collected by clinical project clinicians and recorded using REDCap electronic data capture tools hosted at the Murdoch Children’s Research Institute [21, 22]. Details of the approach (date, site, project) and reason for declining were recorded in a decliners REDCap database. Those who accepted testing completed a test consent form and their details were entered into the main REDCap study database.

### Research survey design

This study employed a pre- (survey 1, S1) and post-test (survey 2, S2) survey to explore the experience of patients having genomic testing as part of their clinical care. Pre- and post-test surveys were initially developed in 2014 for use with patients (and parents of patients) who participated in the Melbourne Genomics demonstration project [6]. Design of the demonstration project surveys was informed by a literature review to identify relevant validated measures and provide insights into custom questions. The draft surveys were reviewed by an expert group comprising Community Advisory Group members [18] and researchers and clinician researchers with relevant expertise. The demonstration project surveys were revised for use in the 2016-2020 program through consultation with clinician researchers, academics and the Community Advisory Group. The survey included a proxy version designed for completion by carers of adults and versions with wording appropriate for a somatic cancer project.

Surveys contained common elements and elements that differed per clinical indication (primarily quality of life measures). Common elements (Supplementary Table 2) included demographics and data capture to evaluate the following domains, using validated measures where possible: experience of counselling; understanding (of test, of result); decision regret (DR) [23]); value of testing; perceived personal control (PPC) [24]. PPC was used as the Genomics Outcomes Scale [25] had not been developed at the time of survey development and the Genetic Counselling Outcomes Scale [26] is not designed for use outside clinical genetic services and, as such, was not applicable across our projects, where testing was offered both within and outside clinical genetic services.

Study-specific ‘value’ question items to assess the value of genomic testing for use in our surveys were generated following an extensive literature review. Content validity of the items was evaluated by clinical and academic professionals and by members of the Melbourne Genomics Health Alliance Community Advisory Group.

### Data collection—research surveys

Participants were asked to complete surveys after consenting to genomic testing (*N* = 1408) and shortly after receiving their results (*n* = 1176). Participants were able to access additional support for completing surveys, including phone interpreters if requested. Patients from the non-Hodgkin Lymphoma (NHL) haematological project were not sent the post-result survey, as genomic results were not available in a time frame to inform clinical management. Surveys are available in Supplementary Materials 2.

### Data analysis

Qualitative data (reasons for declining the offer of testing) were analysed using inductive content analysis[27]. Reasons for declining testing were read through and codes were generated and applied to categorise the responses provided. Codes were then grouped into categories (e.g. insurance, logistics) and categories were then further grouped into general concerns or genomic-specific concerns.

Quantitative survey data were analysed using descriptive statistics in Stata/IC version 15.1 [28]. All survey questions were optional; null responses were excluded from analysis, so denominators vary. Univariable analyses were conducted to assess factors (socio-demographics, test outcomes, clinical project type—rare disease or haematological/malignant) potentially associated with outcomes of interest (survey completion, experience of counselling, understanding, decision regret, perceived personal control) with t-tests and Chi-squared tests used for continuous and categorical variables, respectively. Factors associated with outcomes on univariable analysis were included in multivariable logistic regression analyses. As all analyses were exploratory, no adjustments for multiple testing were applied. *P* values are reported for results where *p* < 0.05. Where reported, 95% confidence intervals (CI) are used.

The experience of counseling was evaluated pre- and post-test. The pre-test counselling survey asked whether respondents had enough information to make decisions, whether they had remaining concerns, and whether they had a chance to ask questions. The post-test survey asked if respondents had enough information to understand their results. Participants could agree, disagree or indicate if they were uncertain. Responses were collapsed into binary outcomes for analysis, with ‘uncertain’ paired with the ‘negative’ response type (e.g. NO remaining concerns compared to YES or UNCERTAIN about remaining concerns).

Pre-test understanding was evaluated using study-specific statements about the types of results the test may return, with 3 response options: True/False/Unsure. Responses were collapsed into correct/incorrect answers. Post-test understanding was assessed by asking about recall of and implications of the result. Responses were cross-checked against test outcomes and classified as correct/incorrect.

Respondents were asked to rate each of the study-specific value statements on a four-point scale (not valuable, neutral, valuable, extremely valuable) or to indicate it was not applicable. Participants with rare disease and bone marrow failure (BMF), where the purpose of testing was to inform diagnosis, were asked 11 value questions. Participants with solid cancers were asked 8 of these questions. Participants with NHL did not complete this survey. Responses to value questions are analysed and reported here for rare disease and BMF, with results from solid cancers previously reported [20]. Responses of ‘not valuable’ were assigned a score of 1, ‘neutral’ and ‘not applicable’ a score of 2, ‘valuable’ a score of 3 and ‘extremely valuable’ a score of 4. ‘Not applicable’ was provided as an option, as we recognised that test outcome (informative or uninformative) might influence peoples’ perception of an item being attained (questions 2, 4-9) or the relevance of an item may be influenced by lifestage (question 3). For the purposes of analysis however, we recognised ‘this was not attained’ (N/A) and ‘I was neutral about this’ (neutral) as equivalent and distinct from responses that assigned a value (‘valuable’, ‘extremely valuable’) or not (‘not valuable’). Mean and standard deviation were calculated per item. Histograms were used to determine the normality of distribution, then t-tests were used to compare mean scores for each item between those with informative and uninformative results. As these value items are not a validated measure, their psychometric structure, validity and scalability were reviewed using item response theory and exploratory factor analysis. Supplementary File 7 provides further details of this assessment. A two-factor structure was determined, and after discussion with the Community Advisory Group, these factors were labeled ‘personal utility’ (factor 1, questions 2-9) and ‘research utility (factor 2, questions 10-11). Imputation was used to derive scores for respondents with a maximum of 1 missing value, for factor 1 only. A mean value score was determined for each factor. As for individual questions, distribution was explored, and t-tests were used to compare mean factor scores from those with and without informative results.

Mean DR scores were calculated for each respondent as per the authors’ instructions [23]: items 2 and 4 were reverse-coded, and scores were converted to a 0-100 scale by subtracting one and multiplying by 25. Scores were then categorised using a previously published system into one of three DR categories: no DR (DR 0), mild DR (DR 1-25), or moderate to high decision regret (DR > 25) [29, 30].

Mean PPC was determined for each time point as per the authors instructions [24] and analysed with paired t-tests. Scores were imputed if fewer than 20% of items were missing. A modified version of the PPC scale, with question 7 about recurrence removed, was used for those with haematological/malignant conditions. Patients with suspected genetic kidney disease were seen in a dedicated renal genetic clinic [31] and, therefore, were asked to complete GCOS rather than PPC.

## Results

### Acceptability of testing

Ninety per cent of those offered genomic testing (1515/1690) accepted, with the highest uptake for solid cancers and the lowest for childhood hearing loss (Table 1).

Seventy-four of the 142 who declined genomic testing gave one or more reasons for their decision (Table 2). The most common reasons for declining testing were a lack of interest in genetic/genomic testing (*n* = 25) and being overwhelmed by/focused on current concerns (*n* = 18). Genetic/genomic testing specific concerns included concerns about data storage and reidentification (*n* = 7) and insurance/financial impacts (*n* = 8).

**Table 2:** Reasons for declining testing.

Reason	*N*
*General concerns/reasons*	
Not interested^a^	25
Overwhelmed/too busy/focused on current concerns	18
Not interested in research^b^	6
Do not believe testing will change care	5
Logistics	5
Do not want a blood test	4
Do not believe condition is genetic	3
Seeking further information from other medical specialists	1
*Genetic/genomic specific concerns*	
Insurance/financial	8
Data collection/storage/concerns about re-identification	7
Uncertain/uncomfortable about genomic testing	4
Childhood autonomy	1

^a^‘Not interested’ covered a range of responses about not being interested in genetic testing.

^b^‘Not interested in research’ covered responses where people did not want testing funded by research or did not want to participate in the aligned research activities.

### Survey response rates and respondent demographics

Survey response rates were 73% (1030/1408) for the pre-test survey (S1) and 53% (626/1176) for the post-test survey (S2); details of S1 respondents have been previously reported [32]. S2 was returned a median of 15 days (IQR 2–35 days) after being sent, or 58 days after receiving results (IQR 22–107 days). Seventy-four respondents who spoke English as an additional language returned S2, with no significant difference observed in S2 completion rates by English language status (55% v 47%, *p* = 0.084). Eight per cent of S2 respondents (50/626) used assistance to complete surveys, including formal assistance from a telephone interpreter (Supplementary Table 3). Considering those sent S2, S2 was more likely to be completed by those tested themselves (adult patients) (adjusted odds ratio [OR] 1.4, 95% CI 1.01–1.93, *p* = 0.042) and those from the highest income bracket (OR 2.18, 95% CI 1.31–3.62, *p* = 0.003) (Supplementary Table 4).

### Respondent experience of counselling

The majority of S1 respondents indicated pre-test counselling provided them with enough information to make a decision about testing (96%; 976/1015) and they had no remaining concerns about the test (89%; 899/1011). A lower proportion of those who spoke English as an additional language indicated they received enough information (88% v 96%, *p* < 0.001) and had no remaining concerns about testing (78% v 91%, *p* < 0.001) (Supplementary Table 5). The related sociodemographic variables ‘born abroad’ and ‘communication assistance required at consent’ showed similar results; level of education did not affect response to these questions (not shown). Respondents with haematological/malignant disease were less likely to have remaining pre-test concerns than those from rare disease projects (8% vs 13%, *p* = 0.005).

Most S2 respondents indicated they received enough information in post-test counselling to understand their result (85%, 499/589). Those from haematological/malignant projects were less likely to agree than those from rare disease projects (75% 132/176 versus 89% 367/413, *p* < 0.0001). There were no differences between respondents by English language status.

### Respondent understanding

After pre-test counselling, two-thirds (67%, 690/1030) of S1 respondents correctly answered all three questions about the types of results the test may return, with a further 19% (191/1030) correctly answering two out of three questions. Those with English as an additional language were less likely to correctly answer each question (Supplementary Table 6). Again, related sociodemographic variables showed similar results whilst level of education did not. Respondents who had incorrectly answered any knowledge question had a higher median time between their last appointment and the time they answered the questions (t = 2.3, *p* = 0.02) than those who had answered all correctly.

After the return of results, S2 asked respondents to recall the outcome of their genomic test. Five per cent of respondents did not answer the question (29/626). Respondents (self or proxy) with haematological/malignant conditions were more likely to leave this question unanswered (9.5% vs 2.5%, *p* < 0.001). Of the respondents who completed the recall question, 86% (511/597) correctly recalled their genomic test result. Most patients reported having received sufficient information from clinicians and genetic counselling to understand their result (85%, 499/589); this was lower in haematological/malignant cohorts (75%, 132/176 vs 89%, 367/413, *p* < 0.001).

### Respondent value

Responses to individual value questions differed based on result type (Table 3). For respondents with informative results, the items of most value were ‘*knowing the cause*’ and providing ‘*information for other members of my family*’. Respondents whose result was uninformative valued having ‘*done everything I can to improve health’*. Those with uninformative results placed the most value on ‘*knowing stored data may be re-examined to find an answe*r’. Value questions related to two factors: ‘personal utility’ (questions 2-9) and ‘research utility’. Mean ‘personal utility’ value was higher for those with informative results (3.16 v 2.68, *p* < 0.0001) and mean ‘research utility’ was slightly higher for those with uninformative results (3.61 v 3.49, *p* = 0.04).

**Table 3:** Respondent values post-result (rare disease and BMF respondents only).

Item	Mean value score (1–4) (standard deviation) *N*	Informative result	Uninformative result	*P* (ttest)
1. No longer requiring ongoing investigations into the condition	2.54 (0.04) *N* = 448	1.84* (0.91) *N* = 170	1.35 (0.84) *N* = 278	*P* < 0.0001
2. Knowing the ‘cause’/explanation for the condition	2.94 (0.05) *N* = 453	2.43* (0.81) *N* = 171	1.65 (1) *N* = 282	*P* < 0.0001
3. Information for my own family planning	2.63 (0.05) *N* = 452	1.95* (0.95) *N* = 170	1.46 (0.94) *N* = 282	*P* < 0.0001
4. Information for other members of my family	3.01 (0.04) *N* = 451	2.34* (0.79) *N* = 170	1.81 (0.96) *N* = 281	*P* < 0.0001
5. Information for treatment/management of the condition	2.84 (0.05) *N* = 448	2.23* (0.9) *N* = 169	1.61 (1.01) *N* = 279	*P* < 0.0001
6. Information regarding prognosis/knowing what to expect in the future	2.88 (0.05) *N* = 445	2.22* (0.90) *N* = 167	1.68 (1.0) *N* = 278	*P* < 0.0001
7. Have had access to the most recent advances in medicine	3.01 (0.05) *N* = 435	2.14* (0.88) *N* = 161	1.93 (0.96) *N* = 274	*P* = 0.024
8. I have done everything I can to improve [my/my child’s/ my relative’s] health	3.12 (0.04) *N* = 431	2.25* (0.82) *N* = 161	2.03 (0.89) *N* = 270	*P* = 0.011
9. Ability to connect with others with the same condition	2.29 (0.04) *N* = 443	1.43* (0.89) *N* = 166	1.21 (0.82) *N* = 277	*P* = 0.008
10. Knowing stored data can contribute to advancing knowledge (research) generally	2.63 (0.61) *N* = 459	2.59 (0.63) *N* = 172	2.65 (0.60) *N* = 287	nsd
11. Knowing stored data may now be examined in more detail to find an answer	2.49 (0.75) *N* = 459	2.37 (0.81) *N* = 169	2.56* (0.71) *N* = 290	*P* = 0.009
Mean ‘personal utility (items 2-9)	2.84 (0.70) *N* = 437	3.12* (0.58) *N* = 164	2.67 (0.71) *N* = 273	*P* < 0.0001
Mean ‘research utility’ (items 10 & 11)	3.56 (0.61) *N* = 456	3.49 (0.62) *N* = 169	3.61* (0.60) *N* = 287	*P* = 0.04

nsd = not significantly different.

*denotes the higher mean.

### Decision regret

Just over half the respondents (54%, 287/534) reported no decision regret (DR). Of those with any DR, 37% reported mild regret (195/534) and 10% reported moderate-high regret (52/534). Factors associated with a higher likelihood of moderate-high DR were: English as an additional language (OR 3.6, CI 1.6–8.3, *P *= 0.003); not receiving enough information at consent (OR 3.76, CI 1.22–11.58, *p *= 0.021), at return of results (OR 2.99, CI 1.40–6.39, *p *= 0.005). Result type (informative, uninformative) was not associated with DR (Supplementary Table 8).

### Perceived personal control

A total of 255 and 155 participants from rare disease and haematological/ malignant projects, respectively, completed the PPC scale at both timepoints. No differences in PPC were observed between pre- and post-test surveys for either group (Table 4) (rare disease mean(SD) S1: 1.05(0.41) v S2: 1.00(0.49); *p* = 0.12; haematological/malignant mean(SD) S1: 1.33(0.45) v S2: 1.33(0.51); *p* = 0.87). When separated by test outcome, PPC increased for those from rare disease projects who received an informative result (mean(SD) S1: 1.06(0.42) v S2: 1.37(0.43); *p* < 0.001, |d | =0.75) and decreased for those without an informative result (mean(SD) S1: 1.05(0.41) v S2: 0.86(0.45); *p* < 0.001, |d | =0.43). Among those with haematological/malignant conditions, no change in PPC was observed after testing, regardless of whether an informative result was received or whether there was a change in treatment.

**Table 4:** Patient reported outcome measure: Perceived Personal Control (PPC).

	Pre-test PPC mean, (SD)	Post-result PPC mean (SD)	Significance *Paired t-tests*
Rare disease			
All respondents			
Informative (*N* = 69)	1.06 (0.42)	1.38 (0.42)	*p* < **0.001 t** = –**6.14**
Uninformative (*N* = 192)	1.05 (0.41)	0.86 (0.45)	*p* < **0.001 t** = **5.65**
Haematological/Malignant			
All respondents			
Informative (*N* = 103)	1.33 (0.8)	1.31 (0.54)	*p* = 0.819 t = 0.23
Uninformative (*N* = 52)	1.36 (0.40)	1.37 (0.46)	*p* = 0.944 t = -0.07

Statistically significant *p*-values are in bold.

## Discussion

As potential applications of genomics expand, evaluation is essential to guide decision-making about when and how to implement genomics in healthcare. By investigating testing under usual clinical governance arrangements - i.e., as close to usual care as possible - and by deliberately targeting inclusion of diverse patients, our results demonstrate equity challenges and offer insights into how patient value might be incorporated into decision-making. We discuss these with reference to their implications for those responsible for making decisions about implementing genomics in healthcare.

Our results indicate disparities in feeling informed and decision regret between those who spoke English as an additional language and those for whom it is their primary language. Translation of genomic terminology is a known challenge [33], but the provision of translated information is not sufficient to overcome barriers to comprehension of genomics [34]. However, before these equity challenges can be addressed by policy-makers, organisations and clinicians, further research is needed to disentangle the extent to which these represent inequities specific to genomic healthcare or are manifestations of general barriers known to impact healthcare equity for people from culturally and linguistically diverse backgrounds [35]. Also of note, across the whole cohort, comprehension of results from somatic testing was lower than for germline testing. This may be explained by the inherent complexity of somatic test results, which may include somatic and germline findings, leading to confusion between the two and informative results may not lead to immediate treatment change [20]. Another potential explanation for this difference is that somatic test results were returned by oncology, rather than genetic professionals. However, genetic professionals may have struggled communicating the relevance of somatic information. Whatever the explanation, both these findings indicate that clinical care processes were clearly not sufficiently supporting all patients to make informed decisions and comprehend genomic test results. Though the solutions to these situations may differ, further testing and evaluation of strategies that support informed decision-making and equitable care are required.

Health technology assessors prefer the use of standardized measures to allow comparison of the value of different technologies [36]. We attempted to employ a common set of measures to determine patient outcomes of genomic testing for a range of conditions spanning cancer and rare diseases, with care provided within or beyond clinical genetics. Others have also grappled with this challenge [19]. As testing in our program was primarily offered outside clinical genetics, we could not use the well-validated GCOS scale [26], nor its revised version [25], which had not been developed at the time of our study. We used Perceived Personal Control (PPC) but found it was not a sensitive scale for those with solid cancers. New measures, such as the P-guide [37], currently under development, will hopefully fill this gap. The set of value questions that we developed and tested here formed a two-factor value scale that did capture value for patients with rare diseases and cancer [20]. This value scale allowed us to move beyond qualitative description [11–13] to capture the patient-derived value of genomic testing quantitatively. We clearly document the different nature of value derived by those with informative and uninformative results, which should reassure decision-makers that there is value even in uninformative results. Much of this value derives from the benefit of contributing to future research, emphasising the importance of establishing pathology systems that allow data sharing of clinically generated data for secondary uses [38] and of scalable approaches to support routine reanalysis of clinically generated genomic data [39].

Decision-makers rightly highlight that evaluation of new technologies must consider potential harms, not only benefits [16]. Our finding that those with uninformative results had lower post-result PPC requires further investigation. The minimum clinically important difference for PPC has not been defined, so we cannot be sure if these statistically different results are clinically meaningful. Reduced PPC after return of results has been previously reported [40], but in that study, it was not associated with result type. Longer-term follow-up using measures with a defined minimum clinically important difference is warranted. Few who declined testing in our cohort did so due to genomic concerns such as data storage, re-identification and insurance or financial implications. At the time of this study, there was a moratorium in place in Australia on the use of genetic testing information in non-community-rated insurance products (e.g. life insurance) [41]. Clear guidelines on storage and re-use of genomic data generated through clinical care and bans on the use of genomic information in insurance decision-making, such as those that exist in Canada [42], should mitigate these. Incorporation of patient perspectives into the design of clinical data sharing systems [32] would also go some way to addressing such concerns. The majority of those who gave a reason for declining stated it was not a priority for them at that time. Service designs that incorporate genomic testing in ‘mainstream’ [43] medical specialty practice provide an opportunity for patients to consider the offer of testing over time as part of their ongoing care with their treating clinician [44], enabling patients to make a decision about testing at the right time for them. Patients in the 100,000 Genomes research project cited concerns about research processes and reluctance to accept end-of-line genomic testing as reasons for declining participation [7], concerns that were not noted in our program. This highlights the value of designs that are as close to the real world as possible to provide insights that support the design of clinical processes for the practical implementation of genomics.

Our evaluation findings should be considered in terms of the strengths and limitations of our study design. We captured uptake of and reasons for declining, clinically indicated genomic testing, to obtain a real-world picture of the acceptability of clinical testing. We also supported completion of evaluation surveys by those with English as an additional language, low literacy, or disabilities that precluded completion of written surveys. These patients are frequently neglected in evaluations of new health technologies, despite calls for health technology assessments to consider equitable access to care alongside maximising health [45]. Our S1 survey completion rates were high, with S2 completion rates equivalent to other published studies. Nevertheless, like others, we did see higher response rates from those in the highest income brackets and, as such, may not have captured the full range of patient perspectives. The absence of measures fit for purpose across the broad range of clinical indications included in our program necessitated the use of different measures for different groups, limiting our sample size for some questions.

International practice in assessing the value of medical tests varies [46], but all rely on research evidence to inform decisions about resource allocation. Our study—as designed—provides evidence about the value patients derive from genomic testing, regardless of test outcomes. Initiatives across the world are striving to improve patient-reported outcomes for genomics; our work contributes to the knowledge of strengths and limitations of current PROMS and introduces a value scale. As genomics is incorporated into health systems, there is an opportunity to address barriers to equity. Our inclusion of a patient group typically overlooked in research—those who speak English as an additional language—highlights challenges with comprehension that cannot be addressed by interpreters alone. The breadth of our work also allowed comparisons across somatic and germline testing, revealing challenges with comprehension of somatic test results. As such, this evaluation of patient experience provides numerous insights to inform future research and the work of decision-makers responsible for implementing genomics in healthcare.

## Supplementary information

Supplementary Materials: Supplementary Tables and Files

Supplementary Materials 2: Master Surveys

## Data Availability

The data that support the findings of this study are available on request from the corresponding author (M.M). The data are not publicly available due to containing information that could compromise research participant privacy.
