# Supplementary material for: Patients offered genomic testing for rare disease and cancer: a real-world evaluation of impact and processes of care
Source: Eur J Hum Genet. 2026 May 19;34(7):985–92. doi: 10.1038/s41431-026-02138-2 (PMC13342248; doi:10.1038/s41431-026-02138-2)
Supplement: Supplementary file 1 — Supplementary Materials: Supplementary Tables and Files [file 41431_2026_2138_MOESM1_ESM.docx]

**Supplementary Files**

Supplementary Table 1: Clinical service design projects funded through the 2016-2019 Melbourne Genomics Health Alliance program

| **Projects** | **Patient Group(s)** | **Test offered** | **Clinicians involved in consent and return of results** |
| --- | --- | --- | --- |
| ***Rare disease projects*** | | | |
| Complex Care (47–49) | Paediatric | Exome sequencing with targeted analysis | Clinical geneticist and genetic counsellor |
| Complex Neurological and Neurodegenerative Diseases (50) | Adult and Adolescent (>12 years) | Exome sequencing with targeted analysis | Neurogeneticist and genetic counsellor |
| Deafness (51) | Paediatric | Exome sequencing with targeted analysis | Clinical geneticist and genetic counsellor |
| Dilated Cardiomyopathy (52) | Adult and Paediatric | Exome sequencing with targeted analysis | Cardiac geneticist and genetic counsellor |
| Immunology (53) | Adult and Paediatric | Exome sequencing with targeted analysis | Immunogeneticist and genetic counsellor |
| Renal Genetics (31) | Adult and Paediatric | Exome sequencing with targeted analysis | Nephrologist, clinical geneticist and genetic counsellor |
| Perinatal autopsy* | Paediatric | Exome sequencing with targeted analysis | Clinical geneticist and genetic counsellor |
| ***Malignant/Hematological projects*** | | | |
| Bone Marrow Failure (54) | Adult and Paediatric | Targeted Panel and  Exome sequencing with targeted analysis | Hematologist and genetic counsellor |
| Non-Hodgkin Lymphoma | Adult | Targeted Panel and  Exome sequencing with targeted analysis  Note: research results not entered into patient records | Hematologist and genetic counsellor |
| Solid Tumors (20) | Adult and Paediatric | Targeted Panel | Oncologist with genomics expertise |
| ***Infectious Diseases**** |  |  |  |
| SuperBugs^+^ (55) |  |  |  |

* Patient surveys not used in these projects

+ This project sequenced bacterial genomes, not human genomes

Supplementary Table 2: Overview of domains included in patient surveys

| **Survey Domain** | **Description** | **S1** | **S2** |
| --- | --- | --- | --- |
| ***Demographics*** |  |  |  |
|  | Age, gender, marital status, education, children, income, postcode, | yes | no |
| ***Counselling*** |  |  |  |
| Pre-test | Study specific questions to assess perception of pre-test counselling (3 items) [information provision, ability to ask questions and remaining concerns] | yes | no |
| Post-test | Study specific question to assess perception of information sufficiency at return of results (1 item) | no | yes |
| ***Understanding*** |  |  |  |
| Pre-test | Study specific questions to assess understanding of topics covered in counselling: 3 items about types of results; 2 about data storage; 1 about additional findings; 2 specific to trios. 3 response options (True, False, Unsure) | yes | no |
| Post-test | Study specific questions to assess recall and understanding of result (2-3 items) | no | yes |
| ***Impact*** |  |  |  |
| Unanticipated consequences^a^ | Study specific question to assess unanticipated consequences of testing (1 question) | no | yes |
| Perceived Personal Control (24) | Nine item questionnaire to assess feelings of control after counselling with three response options (do not agree, somewhat agree, completely agree) allowing numerical scoring. Modified version to patients with haematological/malignant conditions to exclude question 7, which enquires about recurrence. | yes | yes |
| Value | Study specific questions to assess value of the test to the respondent rated as not valuable, neutral, valuable, extremely valuable or not applicable (11 items (8 for solid tumours)) | yes | yes |
| Decision Regret Scale (23) | Five-item questionnaire with five-point Likert Scale which  assesses regret or remorse about a healthcare decision. Categorises patients as having ‘no’, ‘mild’, or ‘moderate-high’ decision regret. | no | yes |

^a^Few comments were made in response to this question so it was not included in the analysis.

Supplementary Table 3: Survey respondent characteristics

| **Characteristic** | Survey 1 responders N=1030  [n(%)] | Survey 2 responders N=626  [n(%)] |
| --- | --- | --- |
| **Gender of survey respondent** |  |  |
| Male | 393 (38) | 187 (30) |
| Female | 620 (60) | 365 (58) |
| Other | 3 (<1) | 1 (<1) |
| Missing | 14 (1) | 73 (12) |
| **Survey type** |  |  |
| Self (adult, adolescent respondent) | 731 (71) | 423 (68) |
| Proxy (parent of paediatric patient, carer) | 299 (29) | 203 (32) |
| **Condition type** |  |  |
| Rare disease | 620 (60) | 437 (70) |
| Malignant/haematological | 410 (40) | 189 (30) |
| **English as a first language** |  |  |
| Yes | 883 (86) | 534 (85) |
| No | 109 (11) | 74 (12) |
| Not stated/inadequately described | 38 (4) | 18 (3) |
| **Assistance to complete surveys^a^** |  |  |
| No | 968 (94) | 576 (92) |
| Yes | 62 (6) | 50^b^ (8) |
| **Income^c^** |  |  |
| Lowest quintile | 206 (20) | 97 (15) |
| Second quintile | 120 (12) | 59 (9) |
| Third quintile | 171 (17) | 91 (15) |
| Fourth quintile | 201 (20) | 106 (17) |
| Fifth quintile | 148 (14) | 98 (16) |
| Missing/prefer not to say | 184 (18) | 175 (28) |

Note: some of the data on S1 respondents was reported in (30) but is provided here for ease of reading. Differences are: gender of survey respondent reported here (not sex of patient); survey type reported here (not patient type adult v child)

^a^ Includes assistance from family members (ie proxy completion)

^b^ Three required phone assistance to complete surveys due to reasons other than language (e.g. writing difficulties due to neurological issues). All three spoke English as a first language.

^c^ Income quintiles are derived from Australian Bureau of Statistics. Census of Population and Housing: Socio-Economic Indexes for Areas (SEIFA), Australia, 2016. https://www.abs.gov.au/ausstats/abs@.nsf/Lookup/by%20Subject/2033.0.55.001~2016~Main%20Features~IRSAD%20Interactive%20Map~16.

Supplementary Table 4: Predictors of survey 2 completion

|  | **Odds Ratio** | **p** | **[95% Conf. Interval]** | |
| --- | --- | --- | --- | --- |
|  |  |  |  |  |
| ***Respondent type*** *- Adult* | 1.40 | 0.042 | 1.01 | 1.93 |
|  |  |  |  |  |
| ***English as an additional language*** | |  |  |  |
| *Yes* | 0.71 | 0.154 | 0.45 | 1.14 |
|  |  |  |  |  |
| ***Income quintile*** | |  |  |  |
| *21-40%* | 1.12 | 0.66 | 0.67 | 1.87 |
| *41-60%* | 1.17 | 0.49 | 0.74 | 1.86 |
| *61-80%* | 1.09 | 0.71 | 0.70 | 1.69 |
| *81-100%* | 2.18 | 0.003 | 1.31 | 3.62 |

Supplementary Table 5: Respondents’ experiences of pre-test counselling according to English language status

|  | Received enough information? | |  | Remaining concerns? | |
| --- | --- | --- | --- | --- | --- |
|  | Yes | No/Unsure |  | Yes/Unsure | No |
| EAL? |  |  |  |  |  |
| Yes | 93 (88%) | 13 (12%)* |  | 23 (22%) | 81 (78%)* |
| No | 849 (97%) | 25 (3%) |  | 82 (9%) | 790 (91%) |
|  |  |  | p < 0.001 |  | p < 0.001 |

EAL= English as an additional language; * p<0.05 chi squared test

Supplementary Table 6: Respondents understanding of possible genomic testing outcomes according to English language status

| Knowledge Statement^ | English as first language | English as an additional language | Chi2, p |
| --- | --- | --- | --- |
|  | **Correct  n (%)** | **Correct  n (%)** |  |
| **1 (test may not provide useful information)** | 725 (84%) | 67 (66%) | χ^2^=19.5, p<0.001 |
| **2 (test may return an uncertain result)** | 718 (84%) | 67 (66%) | χ^2^=18.6, p<0.001 |
| **3 (test may find something that can be passed on in the family)** | 791 (92%) | 85 (84%) | χ^2^=7.1, p=0.008 |

^Knowledge statement:

1. The genomic sequencing test may not identify any gene variants that the doctors think could cause/be associated with the condition. *Answer: True*
2. The genomic sequencing test may identify gene variants that might cause/be associated with the condition, but the doctors are uncertain. *Answer: True*
3. The genomic sequencing test may find gene variants that could be passed on in the family. *Answer: True*

Supplementary File 7: Item Response Theory and exploratory factor analysis of value item questions

The underlying structure, validity and scalability of responses to the 11 item value question set was reviewed using the stata commands *correlate* with options *covariance* and *means*, and *pwcorr*, in line with item response theory. The *factortest* command was used to conduct a Kaiser-Meyer-Olkin (KMO) and Bartlett test of sphericity to ensure sufficient sample size. Exploratory factor analysis (EFA) was conducted using the command *factor* to determine factor structure by grouping related items to aid data summary and structural review. This was done with principal axis factoring to minimise the effect of non-normally distributed data. Items were reviewed for multicollinearity, which was defined as having a correlation to other items of greater than 0.9.(24) Varimax, quartimax, equamax, promax(3 and 2), oblique oblimin (-1,-0.5,0,0.5,1) rotations were all tested to obtained the best resolution. The number of factors to be retained was assed using Eigenvalues and Screeplot. Internal consistency of derived scale was assessed using Cronbach’s *alpha*.

Survey value items were answered by 86% (414/480) of respondents from rare disease and BMF projects. Our sample size exceeded the recommended minimum participant-to-item ratio of 3:1 (37). Pairwise correlation coefficient testing showed no items had inter-correlation >0.8. A Kaiser-Meyer-Olkin measure of sampling adequacy was 0.88. Bartlett’s test of sphericity demonstrated variables were suitably intercorrelated to conduct EFA (χ^2^=842.5, p<0.001).

Screeplot and Eigenvalues suggesting retaining 1 or 2 factors; optimal factor loading was achieved without rotation. The resultant two factor solution mapped items 2-9 to Factor 1, and mapped items 10-11 to Factor 2 (see below). Crohnbach’s alpha analysis of internal validity was 0.90. Item 1 mapped poorly to factor 1 and so was excluded in determining mean factor 1 response. Factor 1 encompasses items related to ‘medical and personal utility’ of testing, with the factor 2 corresponds to ‘social (research) utility’ values.

Factor loading of the value items

|  | Factor 1 | Factor 2 |
| --- | --- | --- |
| 1. No longer requiring ongoing investigations into the condition | 0.4519 | -0.1062 |
| 2. Knowing the 'cause'/explanation for the condition | 0.7789 | -0.1923 |
| 3. Information for my own family planning | 0.5359 | -0.1061 |
| 4. Information for other members of my family | 0.7111 | -0.1006 |
| 5. Information for treatment/management of the condition | 0.837 | -0.1759 |
| 6. Information regarding prognosis / knowing what to expect in the future | 0.8485 | -0.1484 |
| 7. Have had access to the most recent advances in medicine | 0.613 | 0.1114 |
| 8. I have done everything I can to improve [my/my child's/ my relative's] health | 0.5577 | 0.1215 |
| 9. Ability to connect with others with the same condition | 0.5433 | 0.1066 |
| 10. Knowing stored data can contribute to advancing knowledge (research) generally | 0.3301 | 0.6052 |
| 11. Knowing stored data may now be examined in more detail to find an answer | 0.3508 | 0.5861 |

Supplementary Table 8: Predictors of moderate-high decision regret

|  | **Odds Ratio** | **p** | **[95% Conf. Interval]** | |
| --- | --- | --- | --- | --- |
|  |  |  |  |  |
| ***English as an additional language*** | | | | |
| *Yes* | 3.60 | 0.003 | 1.56 | 8.32 |
| ***Insufficient Information at consent*** | | | | |
| *Yes* | 3.77 | 0.021 | 1.22 | 1.60 |
| ***Insufficient information at return of results*** | | | | |
| *Yes* | 2.87 | 0.007 | 1.34 | 6.16 |
| ***Informative result*** | | | | |
| *Yes* | 1.45 | 0.245 | 0.76 | 2.89 |

REFERENCES

See main article

55. Sherry N, Gorrie C, Kwong J, Higgs C, Stuart R, Marshall C, et al. Multi-site implementation of whole genome sequencing for hospital infection control: A prospective genomic epidemiological analysis. The Lancet Regional Health - Western Pacific. 2022 23:100446 doi: 10.1016/j.lanwpc.2022.100446
