# Supplementary material for: Patients offered genomic testing for rare disease and cancer: a real-world evaluation of impact and processes of care
Source: Eur J Hum Genet. 2026 May 19;34(7):985–92. doi: 10.1038/s41431-026-02138-2 (PMC13342248; doi:10.1038/s41431-026-02138-2)
Supplement: Supplementary file 2 — Supplementary Materials 2: Master Surveys [file 41431_2026_2138_MOESM2_ESM.docx]

Supplementary Materials – patient surveys

Participant Survey 1

Thank you for taking the time to complete this survey. This is part one of a two part survey into your perspectives on genomic sequencing. You will be offered part two of the survey after receiving the test results.

Your answers are important to make the case that genomic sequencing has a place in routine/cancer healthcare and to help design the best system for patient care.

This survey should take approximately 30 minutes to complete.

**Before you start:**

There are several words used throughout the survey that we should define for you before you start:

Germline

**Genome:** Everybody has a genome. For the purpose of this survey, the word genome is used to describe all the genes a person has in the cells of their body. Genes contain the instructions that determine many of our characteristics, like our hair colour and eye colour. Some genes are also important for our health. We all have changes in our genes which make us different from each other – these changes are also called ‘variants’.

Cancer

**Genome:** In this study we are examining your cancer cells. For the purpose of this survey, the word genome is used to describe all the genes contained within cancer cells. Genes contain the instructions that determine many of the characteristics of our cancer, like its rate of growth or the response to treatment. Each cancer genome has changes in its genes which make it different from other similar cancers – these changes are also called ‘variants’.

Germline

**Types of variants:** Some variants can cause conditions or increase a person's chance of developing a condition; others may be ‘normal’ variants in the population that seem to have no impact on health and some we do not know the significance of (often called *variants of unknown significance*).

Cancer

**Types of gene variants:** In cancer, some variants can change the way a cancer behaves; others may be ‘normal’ variants in the cancer that seem to have no impact on cancer growth or response to treatment and some we do not know the significance of (often called variants of unknown significance).

**Genomic sequencing:** There are several types of genomic sequencing tests, including whole genome, whole exome and panel tests. These tests capture information from many or even all genes at the same time. This information can then be looked at (analysed) to check for any changes (variants) that may be present. The analysis can look at different numbers of genes. In this study, your/your child’s genes are being tested to look for variants that can cause [germline]/are associated with [cancer] your [patient]/their [parent, proxy] condition.

If you have any questions or concerns about the survey please contact [contact details for project and site]:

Date completed (DDMMYY):

Section 1: Information about you

*Please complete the following section to tell us about* ***yourself*** *(the person completing the survey):*

Are you completing this survey as…

- An individual who is having a genomic sequencing test
- A parent of a child who is having a genomic sequencing test Age of child tested: __________
- A carer of a relative who is having a genomic sequencing test (please tell us your relationship to the person you care for e,g. partner, sibling, child) _______________

1. How old are you? __________ years
2. What gender are you?

⬜ Female ⬜ Male ⬜ Other

1. What is your current marital status?

⬜ Married ⬜ Divorced/separated

⬜ De facto (living with a partner) ⬜ Never married

⬜ Widowed ⬜ Other (please specify): ____________________

1. What is your highest level of education?

⬜ Year 11 or below ⬜ Bachelor degree

⬜ Year 12 or equivalent ⬜ Graduate Diploma/Certificate

⬜ Certificate ⬜ Post-graduate degree

⬜ Diploma/Advanced diploma ⬜ Other (please specify): ____________________

1. For “adult” surveys only [not for adolescents]
   1. Do you have any biological children?

- No
- Yes, I have ________ child(ren)

Please list the ages of your children

Child 1__________ Child 4 __________ Child 7 __________

Child 2__________ Child 5 __________ Child 8 __________

Child 3__________ Child 6 __________ Child 9 __________

- 1. Which statement best describes you? [not included in cancer surveys]
- I have not yet had children and am not thinking about having children at this time
- I have not yet had children but I am planning to have children in the near future
- I am still having children
- I have already had my children and do not plan to have any more
- I have not and will not have children

Optional comment: ________________________________________________________________________________________________________________________________________________________________________

1. [For “parent” surveys only] [not included in cancer surveys]
   1. How many living children do you have? I have __________children

Please list the ages of your children

Child 1__________ Child 4 __________ Child 7 __________

Child 2__________ Child 5 __________ Child 8 __________

Child 3__________ Child 6 __________ Child 9 __________

- 1. Are you planning to have any (more) children?
- Yes
- Unsure
- No

Optional Comment:

__________________________________________________________________________________________________________________________________________________________________________

1. If you were to have another child, how concerned would you be about the condition occurring again? [For “parent” surveys only] [not included in cancer surveys]

⬜ Not concerned

⬜ Moderately concerned

⬜ Extremely concerned

1. Please indicate your gross family income (i.e. the combined income of everyone in the household), before tax and other deductions are taken out, during the last financial year.

⬜ Less than $4,900 per year ⬜ $60,000 to $69,999 per year

⬜ $5,000 to $9,999 per year ⬜ $70,000 to $79,999 per year

⬜ $10,000 to $19,999 per year ⬜ $80,000 to $89,999 per year

⬜ $20,000 to $29,999 per year ⬜ $90,000 to $119,999 per year

⬜ $30,000 to $39,999 per year ⬜ $120,000 to $149,999 per year

⬜ $40,000 to $49,999 per year ⬜ $150,000 or more per year

⬜ $50,000 to $59,999 per year ⬜ Prefer not to answer

1. What is your postcode? _________
2. Do you have private health insurance? _________

Section 2: Your decisions about the genomic sequencing test

Before agreeing to the genomic sequencing test, you met with a health professional and were given information about genomic sequencing. At this consultation you were asked to make 2 decisions.

Decision 1: Whether to have the test

Decision 2: Whether to share your re-identifiable data from the test for research

1. Below is a list of ways in which you might have received info about the test. Please tell us how useful you found each information source. If you didn’t use this source, tick not applicable. [not included in cancer surveys]

|  | Extremely valuable | Valuable | Neutral | Not valuable | Not Applicable |
| --- | --- | --- | --- | --- | --- |
| Conversation with a doctor (e.g. haematologist, neurologist) |  |  |  |  |  |
| Conversation with a genetic counsellor / genetics doctor |  |  |  |  |  |
| Printed information |  |  |  |  |  |
| Online information |  |  |  |  |  |

Please comment (if you used any other sources of information, please list them here)

___________________________________________________________________________________________________________________________________________________________________________________________________________________________________________________

Section 2.1: Your decision to agree to the test

The following questions are about the **information** you received before agreeing to the test.

1. Do you feel you received enough information about the test before you agreed to it?

⬜ Yes ⬜ No ⬜ Unsure

If No or Unsure, please comment below:

___________________________________________________________________________________________________________________________________________________________________________________________________________________________________________________

1. Did you have the chance to ask all the questions you had about the test before you agreed to it?

⬜ Yes ⬜ No ⬜ Unsure

If No or Unsure, please comment below:

___________________________________________________________________________________________________________________________________________________________________________________________________________________________________________________

1. After receiving information and having the opportunity to ask questions, do you have any remaining concerns about the test?

⬜ Yes ⬜ No ⬜ Unsure

If Yes or Unsure, please comment below:

___________________________________________________________________________________________________________________________________________________________________________________________________________________________________________________

1. The following questions are about your **reasons** for agreeing to the genomic sequencing test and expectations of genomic sequencing.

|  | Extremely important | Important | Neutral | Unimportant | Extremely unimportant |
| --- | --- | --- | --- | --- | --- |
| To find a “cause”/explanation for the condition/cancer |  |  |  |  |  |
| To obtain information for my own family planning [not cancer] |  |  |  |  |  |
| To obtain information for other members of my family |  |  |  |  |  |
| To obtain information for treatment or management of the condition/cancer |  |  |  |  |  |
| To obtain information regarding prognosis/what to expect in the future |  |  |  |  |  |
| To avoid ongoing investigations |  |  |  |  |  |
| To allow me to connect with others with the same condition |  |  |  |  |  |
| To contribute to research |  |  |  |  |  |

Comment (Please comment about the choices listed above or any other things that you hope to learn from the genomic sequencing test):

___________________________________________________________________________________________________________________________________________________________________________________________________________________________________________________

1. [Hereditary and Bone Marrow Failure projects]
   1. Please indicate how likely you think it is that genomic sequencing will find the cause of your /your child’s condition (*please tick one only*):

- Highly likely
- Likely
- Equal chance of finding the cause and not finding the cause
- Unlikely
- Highly unlikely
- I don’t know

1. [Cancer projects]
   1. What do you think the chance is that that the genomic sequencing test result will lead to a change in your/your child’s treatment?

- 0 - 24% chance
- 25 – 50% chance
- 51 – 75% chance
- More than 75% chance
- I don’t know
  1. How likely do you think it is that you have developed cancer as a result of an inherited family cancer gene?
- Highly unlikely
- Unlikely (but possible)
- Equally likely as unlikely
- Likely
- Highly likely
- Unsure
  1. How concerned are you about other family members developing cancer like you/your child?
- Not concerned
- Slightly concerned
- Concerned
- Highly concerned

1. We are interested in how the genomic sequencing test may be **valuable** to you and your family. Please indicate how valuable the following might be to you. We have provided space for comments.

|  | Extremely valuable | Valuable | Neutral | Not valuable | Not Applicable |
| --- | --- | --- | --- | --- | --- |
| To find a “cause”/explanation for the condition/cancer |  |  |  |  |  |
| Information for my own family planning [not cancer] |  |  |  |  |  |
| Information for other members of my family |  |  |  |  |  |
| Information for treatment / management of the condition |  |  |  |  |  |
| Information regarding prognosis/ knowing what to expect in the future |  |  |  |  |  |
| The knowledge that I won’t have to have other investigations for the condition [not cancer] |  |  |  |  |  |
| The fact that I am taking advantage of the most recent advances in medicine |  |  |  |  |  |
| The idea that I’m doing everything I can to improve my health |  |  |  |  |  |
| To connect with others with the same condition [not cancer] |  |  |  |  |  |
| The idea that we are contributing to research |  |  |  |  |  |

Comments __________________________________________________________________________________________________________________________________________________________________

1. We are interested to know the value that you place on the answers that may come from genomic testing. Suppose that the genomic test provides you with more certain answers about the cause of the condition, which may provide information to guide the management and/or treatment of your condition. Now suppose that you live in a country like the United States where people have to pay for their test.

Please indicate the MAXIMUM amount you would be willing to pay for this genomic test:

**Note: You will not be asked to actually pay for the test in this study.**

- 1. What is the MOST you would be prepared to pay for this genomic test?

| ⬜ $500  ⬜ $1000  ⬜ $1500  ⬜ $2000  ⬜ $2500  ⬜ $3000  ⬜ $3500  ⬜ $4000  ⬜ $4500 | ⬜ $5000  ⬜ $5500  ⬜ $6000  ⬜ $6500  ⬜ $7000  ⬜ $7500  ⬜ $8000  ⬜ Other (please specify): $________________  ⬜ I would NOT be willing to pay for this genomic test |
| --- | --- |

If you gave an amount you would be willing to pay to the above, please answer this question

- 1. If you WOULD be willing to pay for this genomic test, how certain are you that you would pay the amount you indicated?
- Definitely sure
- Reasonably sure
- Not so sure

If you said you would not be willing to pay, please answer this question

- 1. If you WOULD NOT be willing to pay for this genomic test, please tell us why:
- The information is of no value to me and my family
- Someone else should pay for it (e.g. the Government)
- I cannot afford it
- Other (please specify): ________________________________________________________________

Section 2.2: Your decision about sharing data for research

[Excluded - reported in Martyn et al 2024 “Secondary use of genomic data: patients’ decisions at point of testing and perspectives to inform international data sharing” European Journal of Human Genetics 32: 717-742 doi: 10.1038/s41431-023-01531-5]

Section 3: Your understanding of genomic sequencing

1. The following statements are related to your understanding of the genomic sequencing test. Your responses will help us improve the information we provide about genomic sequencing in the future.

|  | True | False | Unsure |
| --- | --- | --- | --- |
| The test may not find any gene variants that the doctors think could cause [germline]/be associated with the condition/cancer |  |  |  |
| The test may find gene variants that might cause/be associated with the condition/cancer, but the doctors are uncertain. |  |  |  |
| The test may reveal gene variants that can cause other unrelated conditions that may develop in the future |  |  |  |
| The test may find gene variants that could be passed on in the family. |  |  |  |
| The data from the genomic sequencing test cannot be stored and looked at in the future - a new test is required each time. |  |  |  |
| Anonymised data from the test can be used for the purpose of advancing knowledge (research). |  |  |  |

1. Please select the correct statement by placing a tick in only one of the three boxes below. As part of the genomic sequencing test:

- One gene will be analysed (looked at)
- Many genes will be analysed (looked at)
- All genes will be analysed (looked at)

Section 4: Living with the condition/cancer

1. The genomic test has been offered in relation to a particular condition/cancer. Please indicate how much you agree with each statement.

| *Please make sure you tick one box*  *for each statement* | Do not agree | Somewhat agree | Completely agree |
| --- | --- | --- | --- |
| I think I understand for which condition the genomic sequencing test was offered | 0 | 1 | 2 |
| I feel I know the meaning of the condition/cancer for my family’s future and me. | 0 | 1 | 2 |
| I think I know what caused the condition/more about the cancer. | 0 | 1 | 2 |
| I feel I have the tools to make decisions that will influence my future. | 0 | 1 | 2 |
| I feel I can make a logical evaluation of the various options available to me in order to choose one of them. | 0 | 1 | 2 |
| I feel I can make decisions that will change my family’s future [germline]/ impact my family [cancer]. | 0 | 1 | 2 |
| I feel there are certain things I can do to prevent the condition from recurring. [not cancer] | 0 | 1 | 2 |
| I feel I know what to do to ease the situation. | 0 | 1 | 2 |
| I think I know what my next steps should be. | 0 | 1 | 2 |

(Perceived Personal Control questions from Berkenstadt et al, 1999)

[The rest of this section contains questions on utilisation of health services and standardised measures for quality of life and patient reported outcome measures. These varied project to project]

The questions below will help us understand whether the genomic sequencing test helps you and your family with the condition for which testing was offered.

1. Please tell us about your current use of healthcare services. Which health professionals did you see at least once in the last year, approximately how many visits did you have with them and how many of those occurred in the public health system (you did not pay for privately) [Germline. Complex Neurological and Neurodegenerative Diseases project only]

| Health professional | Seen in last 12 months? | Number of times seen in last 12 months | % public |
| --- | --- | --- | --- |
| Medical specialists | | |  |
| neurologist | ⬜ Yes If yes ->  ⬜ No | ______ |  |
| neuropsychiatrist | ⬜ Yes If yes ->  ⬜ No | ______ |  |
| psychiatrist | ⬜ Yes If yes ->  ⬜ No | ______ |  |
| clinical geneticist | ⬜ Yes If yes ->  ⬜ No | ______ |  |
| other __________ |  | ______ |  |
| Allied health professionals | | |  |
| dietician | ⬜ Yes If yes ->  ⬜ No | ______ |  |
| genetic counsellor | ⬜ Yes If yes ->  ⬜ No | ______ |  |
| neuropsychologist | ⬜ Yes If yes ->  ⬜ No | ______ |  |
| occupational therapist | ⬜ Yes If yes ->  ⬜ No | ______ |  |
| physiotherapist | ⬜ Yes If yes ->  ⬜ No | ______ |  |
| podiatrist | ⬜ Yes If yes ->  ⬜ No | ______ |  |
| psychologist | ⬜ Yes If yes ->  ⬜ No | ______ |  |
| social worker | ⬜ Yes If yes ->  ⬜ No | ______ |  |
| speech pathologist, | ⬜ Yes If yes ->  ⬜ No | ______ |  |
| other __________ |  |  |  |

1. Do you currently have access to social care supports e.g. respite care, home modification assistance, help in the home [personal carers, germline. Complex Neurological and Neurodegenerative Diseases project only]

⬜ Yes, government funded ⬜ Yes, self-funded ⬜ No

If yes, which services ________________________________________________________________

1. How many hours per week of care do you provide for your relative? __________/week [carers only]
2. Are you currently in paid employment [carers only]

⬜ Yes, working _____ hours/week ⬜ No

1. Do your caring duties affect your employment? [carers only]

⬜ Yes, choice of job ___________

⬜ Yes, hours I would work X/hours per week

⬜ No

Section 5: Genomic sequencing tests in the future [not cancer]

[Excluded – reported in Martyn et al 2024 “Evaluation of a two-step model of opportunistic genomic screening” European Journal of Human Genetics 32:656-664 doi: 10.1038/s41431-024-01592-0]

Section 6: Data Storage in the Future

[Excluded – reported in Martyn et al 2024 “Secondary use of genomic data: patients’ decisions at point of testing and perspectives to inform international data sharing”. European Journal of Human Genetics 32: 717-742 doi: 10.1038/s41431-023-01531-5]

☺ Thank you for completing this survey! ☺

Participant Survey 2

Thank you for taking the time to complete this survey. This is part two of a two [germline & non-Hodgkin’s lymphoma]/ three [Solid tumours] part survey into your perspectives on genomic sequencing. You were offered part one of the survey before receiving the test results.

Your answers are important to make the case that genomic sequencing has a place in routine healthcare and to help design the best system for patient care.

This survey should take approximately X minutes to complete.

**Before you start:**

There are several words used throughout the survey that we should define for you:

Germline

**Genome:** Everybody has a genome. For the purpose of this survey, the word genome is used to describe all the genes a person has in the cells of their body. Genes contain the instructions that determine many of our characteristics, like our hair colour and eye colour. Some genes are also important for our health. We all have changes in our genes which make us different from each other – these changes are also called ‘variants’.

Cancer

**Genome:** In this study we are examining your cancer cells. For the purpose of this survey, the word genome is used to describe all the genes contained within cancer cells. Genes contain the instructions that determine many of the characteristics of our cancer, like its rate of growth or the response to treatment. Each cancer genome has changes in its genes which make it different from other similar cancers – these changes are also called ‘variants’.

Germline

**Types of variants:** Some variants can cause conditions or increase a person chance of developing a condition; others may be ‘normal’ variants in the population that seem to have no impact on health. There are some variants for which we do not yet know whether they contribute to a condition (often called variants of unknown significance).

Cancer

**Types of gene variants:** In cancer, some variants can change the way a cancer behaves; others may be ‘normal’ variants in the cancer that seem to have no impact on cancer growth or response to treatment and some we do not know the significance of (often called variants of unknown significance).

**Genomic sequencing:** There are several types of genomic sequencing tests, including whole genome, whole exome and panel tests. These tests capture information from many or even all genes at the same time. This information can then be looked at (analysed) to check for any changes (variants) that may be present. The analysis can look at different numbers of genes. In this study your/your child’s genes are being tested to look for variants that can cause [germline]/ be associated with [cancer] your [patient]/their [parent, proxy] condition.

If you have any questions or concerns about the survey, please contact:

Date completed (DDMMYY):

Section 1: The genomic sequencing result

1. Which of the following best describes the genomic sequencing result? (Please tick one only) [Germline only]

|  | | |
| --- | --- | --- |
| One or more gene variants were found that are the likely cause of the condition | ☐ | ® Answer Q2a and then go to Q5 |
| No gene variants were found that are the likely cause of the condition | ☐ | ® Answer Q2b and then go to Q5 |
| One or more gene variants were found but it is not clear whether any of these could cause the condition | ☐ | ® Answer Q2c and then go to Q5 |
| I’m not sure what the result was (please comment):  _______________________________________________________ | ☐ | ® Go to Q5 |
| Other (please specify):  _______________________________________________________ | ☐ | ® Go to Q5 |

1. [Germline only]
   1. Does the test result have any implications for relatives?

_____________________________________________________________________________________________________________________________________________________________________________________________________________________________________________

- 1. What do you believe the genomic sequencing result tells you about the cause of the condition? (*tick as many as you think apply*)
- The condition is not genetic
- The condition may be genetic but the cause was not detected by the test
- Don’t know (please comment):________________________________
  1. What does this uncertain result mean to you and your family?

_____________________________________________________________________________________________________________________________________________________________________________________________________________________________________________

1. [Cancer only]
   1. What was the result of the genomic sequencing test?

- There were no gene variants found (go to Q4)
- One or more gene variants were found but it is not clear whether the doctor can act on these (go to Q3b)
- One or more gene variant(s) were found, which the doctors can act on (go to Q3b)
- I am unsure what my result was (go to Q4)
  1. In which cells were the gene variant(s) found? (tick all that apply)

⬜ Within the cancer cells (that cannot be inherited)

⬜ Within normal body cells (that can be inherited). If yes, please answer 3c below


⬜ One or more gene variant was found in body cells but it’s not clear if these influenced my/my child’s cancer

⬜ One or more gene variants were found which have influenced my/my child’s cancer

1. What do you think this test result means for your relatives, if anything? [Cancer only]

___________________________________________________________________________________________________________________________________________________________________________________________________________________________________________________

For all

- 1. We are interested in knowing how valuable the genomic sequencing test has been for you and your family [germline] / you/your child [cancer]. Please indicate how valuable the following are to you, or tick “Not applicable” if the test has not provided you with this. We have provided space for comments.

|  | Extremely valuable | Valuable | Neutral | Not valuable | Not applicable |
| --- | --- | --- | --- | --- | --- |
| Ongoing investigations into the condition no longer necessary |  |  |  |  |  |
| Knowing the “cause”/explanation for the condition |  |  |  |  |  |
| Information for my own family planning |  |  |  |  |  |
| Information for other members of my family |  |  |  |  |  |
| Information for treatment / management of the condition |  |  |  |  |  |
| Information regarding prognosis/ knowing what to expect in the future |  |  |  |  |  |
| Have had access to the most recent advances in medicine |  |  |  |  |  |
| I have done everything I can to improve my/my child’s health |  |  |  |  |  |
| Ability to connect with others with the same condition |  |  |  |  |  |

- 1. Please discuss the main benefits that your test results have had for you e.g. treatment, psychological, emotional, social, family-related, knowing about risks of cancer for other family members

_____________________________________________________________________________________________________________________________________________________________________________________________________________________________________________
_______________________________________________________________________________

1. Genomic testing makes it possible to store data so it can be looked at again, either to continue to try and find an answer or for future research. How valuable is it that your/your child’s data may now be examined in more detail to find an answer?

- Extremely valuable
- Valuable
- Neutral
- Not valuable
- Not applicable

Please comment:

___________________________________________________________________________________________________________________________________________________________________________________________________________________________________________________

1. How valuable is it to you that your/your child’s stored data can contribute to advancing knowledge (research) generally

- Extremely valuable
- Valuable
- Neutral
- Not valuable
- Not applicable

Please comment:

___________________________________________________________________________________________________________________________________________________________________________________________________________________________________________________

1. Do you believe that your cancer treatment has or will be impacted by undergoing this test? [Cancer Only]

___________________________________________________________________________________________________________________________________________________________________________________________________________________________________________________

1. Has anything happened as a result of having the test that you hadn’t expected? [All]

⬜ Yes ⬜ No

If yes, please comment below:

___________________________________________________________________________________________________________________________________________________________________________________________________________________________________________________

1. The quality of the DNA from your tumour wasn’t good enough to get a sample, so we were unable to do this test. When you were told about the test, do you remember being told that this might occur? [Cancer sample fails only]

⬜ Yes ⬜ No ⬜ Unsure

Section 2: Receiving the result

1. During the consultation in which you received the test results, do you feel you received enough information to understand the result?

⬜ Yes ⬜ No ⬜ Unsure

If no or unsure, please comment. What additional information would have been helpful?

___________________________________________________________________________________________________________________________________________________________________________________________________________________________________________________

1. Please think about the decision you made about agreeing to the genomic sequencing test after talking to the health care professional. Please show how you feel about these statements by selecting a number from 1 (strongly agree) to 5 (strongly disagree)

|  | Strongly Agree | Agree | Neither Agree Nor Disagree | Disagree | Strongly Disagree |
| --- | --- | --- | --- | --- | --- |
| It was the right decision | 1 | 2 | 3 | 4 | 5 |
| I regret the choice that was made | 1 | 2 | 3 | 4 | 5 |
| I would go for the same choice if I had to do it over again | 1 | 2 | 3 | 4 | 5 |
| The choice did me a lot of harm | 1 | 2 | 3 | 4 | 5 |
| The decision was a wise one | 1 | 2 | 3 | 4 | 5 |

(Decision Regret Scale questions from Brehaut et al, 2003)

Please comment

__________________________________________________________________________________________________________________________________________________________________

Section 3: Living with the condition

The questions below will help us understand whether the genomic sequencing test helps you and your family with the condition you’ve been offered testing for.

1. The genomic test has been offered in relation to a particular condition. In relation to this condition, please indicate how much you agree with each statement.

| *Please make sure you tick one box*  *for each statement* | Do not agree | Somewhat agree | Completely agree |
| --- | --- | --- | --- |
| I think I understand for which condition the genomic sequencing test was offered | 0 | 1 | 2 |
| I feel I know the meaning of the condition for my family’s future and me. | 0 | 1 | 2 |
| I think I know what caused the condition. | 0 | 1 | 2 |
| I feel I have the tools to make decisions that will influence my future. | 0 | 1 | 2 |
| I feel I can make a logical evaluation of the various options available to me in order to choose one of them. | 0 | 1 | 2 |
| I feel I can make decisions that will change my family’s future. | 0 | 1 | 2 |
| I feel there are certain things I can do to prevent the condition from recurring. | 0 | 1 | 2 |
| I feel I know what to do to ease the situation. | 0 | 1 | 2 |
| I think I know what my next steps should be. | 0 | 1 | 2 |

(Perceived Personal Control questions from Berkenstadt et al, 1999)

1. Has the test had an impact on whether or not you want more children? [For “parent” surveys only]

⬜ Yes ⬜ No

If Yes, how?

- Planning more children (we have ____ children and would now like to have ____ children)
- Planning no more children (we wanted ____ children but will now complete our family with ____ children)

Please tell us how the test has impacted on your plans for your family / family planning:

____________________________________________________________________________________________________________________________________________________________________________________________________________________________________________________________________________________________________________________________________

Section 4: Genomic sequencing tests in the future [not cancer]

[Excluded- reported in Martyn et al 2024 “Evaluation of a two-step model of opportunistic genomic screening” European Journal of Human Genetics 32:656-664 doi: 10.1038/s41431-024-01592-0]

☺ Thank you for completing this survey! ☺
